# A Qualitative Study of Medical Oncologists’ Experiences of Their Profession and Workforce Sustainability

**DOI:** 10.1371/journal.pone.0166302

**Published:** 2016-11-30

**Authors:** Alex Broom, W. K. Tim Wong, Emma Kirby, David Sibbritt, Deme Karikios, Rosemary Harrup, Zarnie Lwin

**Affiliations:** 1 School of Social Sciences, UNSW Australia, Sydney, New South Wales, Australia; 2 Faculty of Health, University of Technology, Sydney, New South Wales, Australia; 3 NHMRC Clinical Trials Centre, University of Sydney, Sydney, New South Wales, Australia; 4 Nepean Hospital, Sydney, New South Wales, Australia; 5 Royal Hobart Hospital, Hobart, Tasmania, Australia; 6 Royal Brisbane & Women’s Hospital, Brisbane, Queensland, Australia; 7 University of Queensland, Brisbane, Queensland, Australia; University of South Australia, AUSTRALIA

## Abstract

**Background:**

Medical oncology is a steadily evolving field of medical practice and professional pathway for doctors, offering value, opportunity and challenge to those who chose this medical specialty. This study examines the experiences of a group of Australian medical oncologists, with an emphasis on their professional practice, career experiences, and existing and emerging challenges across career stages.

**Methods:**

In-depth qualitative interviews were conducted with 22 medical oncologists, including advanced trainees, early-career consultants and senior consultants, focusing on: professional values and experiences; career prospects and pathways; and, the nexus of the characteristics of the profession and delivery of care.

**Results:**

The following themes were emergent from the interviews: the need for *professional reinvention* and the pressure to perform; the importance, and often absence, of *mentoring* and *feedback loops*; the *emotional labour* of oncology; and, the impact of cascading *workload volume* on practice sustainability.

**Conclusions:**

Understanding professional experiences, career trajectories and challenges at the workforce level are crucial for understanding what drives the oncological care day-to-day. The results indicate that there are considerable potential tensions between the realities of professional, workforce demands and expectations for patient care. Such tensions have real and significant consequences on individual medical oncologists with respect to their futures, aspirations, satisfaction with work, caring practices, interactions with patients and potentially therapeutic outcomes.

## Introduction

The emergence of chemotherapy as an effective treatment for cancer in the late 1940s led to the development of medical oncology as a medical specialty in the 1970s [1]. In Australia, medical oncology is a well-established speciality with its peak representative body–Medical Oncology Group of Australia (MOGA)–formed in the late 1970s [2]. Over the subsequent thirty plus years, there have been notable changes in the expectations and demands of the medical oncology workforce itself. For example, medical oncologists have assumed a central role in multidisciplinary cancer care, often serving as the ‘patient interface’ between other oncology healthcare professionals and patients [3], and are crucial in meeting the demand for, and the delivery of, quality clinical cancer care as well as psychosocial care for their patients. It is therefore unsurprising that medical oncology is, like many other areas of medicine, steadily evolving in terms of the character of the work and the structural pressures on the profession as a whole (see also [4, 5]).

Changes in medical oncology work, and in turn, the profession, have raised questions pertaining to the adequacy of, and viability of, the workforce in meeting anticipated clinical demands. Workforce surveys conducted in Australia [6–8] and internationally [5, 9–11] have raised considerable issues around the state of the profession in terms of its capacity to meet anticipated needs by describing the situation as a “looming threat to quality cancer care” ([8], p.32). Hitherto workforce studies have provided a snapshot perspective, without exploration of the nuanced experiences of individual clinicians. Thus little is known about the (evolving) experience of day-to-day medical oncology work, and importantly, the nexus of structural pressures, professional values, career ambitions and clinical practice.

### Intensification, insecurity and professional reinvention

Medicine is traditionally held as a ‘safe’ career choice as it has offered security in terms of job opportunities [12]. One of the appeals of medical oncology as a medical specialty has been the training/job opportunities for those who qualify [13]. Yet as with other areas of medicine, competition, contraction in employment opportunities (despite patient volumes), and fractional jobs have created a more challenging work environment, which is altering workforce experiences and resulting in greater intensification of the medical oncology labour process (cf. [14]). Work intensification, in this context, manifests in higher and higher *benchmarks for success*, and the emphasis on attaining distinction through achievements in order to be sustainable in a perceived competitive market with diminished opportunities (cf. [15]). In this regard, the assumed security in achieving a medical specialty career is no longer guaranteed as a result of changes to workplace demands, a situation reflected in other medical specialties (see [16, 17]).

There are broader patterns in workforce experiences that have been explored within the scientific literature. For example, the emergence of *insecurity* and *intensification* within the traditionally ‘safe’ professions (e.g. medicine) has been a feature of workplace relations over the course of the late 20^th^ and now early 21^st^ centuries. This has been analysed less in medicine–or medical oncology–than in other fields (cf. [18]) and thus presents as a potentially valuable approach to understanding what is occurring in medicine and in medical oncology more specifically. In the context of the medical oncology profession where changes in its structure and everyday practices are happening, there is an imperative for its practitioners to be flexible and reconfigure a different, if not new, approach–professional reinvention–to their practices and future professional directions.

*Professional reinvention* [19] has become a common requirement across many different spheres of work in modern societies, a process that has been linked to the pressures and consequences of work in modern capitalism [20]. That is, a problematic mix of flexibility, and short-term (material successes) without the rewards of longer-term commitments and loyalties (whether centred on quality of care, or institutional functioning). The contemporary characteristics of flexibility–here and in this study articulated as ‘professional reinvention’–offer both opportunities and oppressive potential. The shifting expectations and capabilities required in the contemporary workforce has also been explored by social and behavioural scientists [21], including the issue of reinvention, the emerging insecurities of younger and/or the new generation of workers [22]–in this case, doctors. This is embedded in, or perhaps confounded by, the broader dynamics of economic reform, privatisation and the widening gap between funding and demand in the public sector in the context of global (and local) austerity. In Australia, funding for health services, and cancer services more specifically, has simply not kept up with demand [23, 24]. Moreover, whilst medical training previously meant a virtual guarantee of a successful and fruitful career–albeit high pressured and competitive–this informal social contract has become increasingly challenged by the realities of practice and career trajectories.

### Towards an understanding of professional pressures and evolutions

While we acknowledge the plenitude of works exploring the challenges and pressures of medical training and careers (cf. [25]), we argue that the themes outlined here provide important new insights into the (evolving) nexus of structural/service strains, funding models, political landscapes, professional change and micro-level interactions. That is, the experience of a medical oncology career reveals many of the broader tensions within contemporary medical labour, including ideals versus pragmatics. In the context of oncology such dilemmas are acutely important, feeding into the character of care and support cancer patients receive. Understanding dynamics at a workforce level is one important facet of fostering caring relations and positive therapeutic outcomes [26, 27]. Building on the concerns identified from survey-based studies around professional sustainability and cascading workload volume in medical oncology (e.g., [7]), the aim of this study is to explore the experiences and expectations of a group of Australian medical oncologists, and the implications for the present and future viability of care within oncological settings.

## Methods

### Data collection and sample

Ethics approval was received from The University of Tasmania Human Research Ethics Committee (Ref: H0014781), and all participants provided written informed consent. Employing an inductive approach, data was collected through in-depth qualitative interviews with 22 medical oncologists at different stages of their careers and working in both major city and inner regional settings [28]–participant characteristics are contained in Table 1. The participants were recruited through MOGA membership lists. An initial email invitation was sent to New South Wales MOGA members with a participant information sheet detailing the aim of the study and participation requirements. Potential participants were asked to contact a member of the research team for further clarification and/or to register their interest to participate. Informed by snowball and convenience sampling strategy [29], participants were also recruited through colleagues who either knew of or were participating in the study. Everyone who indicated an interest to participate was interviewed. Following completion and initial analysis of the interviews, the researchers agreed that data saturation had been reached–namely, we had reached the point when it was likely that no new themes would emerge relating to the focal areas of the study [30]. Each interview lasted between 60 and 90 minutes, was digitally audio-recorded and transcribed in full by a professional transcribing company.

**Table 1:** Participant characteristics (n = 22).

**Sex**	Female	9
	Male	13
**Career stage**	Advanced trainee	8
	Early-career consultant	6
	Senior consultant	8
**Work Location**	City	19
	Regional (including all categories as defined by the Australian Government Department of Health [31])	3
**Years in medical practice**	0–2 years	8
	3–5 years	6
	6–10 years	3
	11–20 years	2
	21+ years	3

### Analysis

This research, our conceptual framework, methodology and analytic approach therein was informed by interpretivist traditions in sociology. This approach broadly seeks to observe coherences and contrasts within participants’ subjective interpretations of their field, profession and everyday practices. Given the aim of the analysis was to achieve a comprehensive and detailed understanding of the experiences and perspectives of medical oncologists, and locate these within broader underlying themes, we conducted thematic analysis of the full interview transcripts to reveal patterns within and across the data set, using NVivo 11™ software as a data management tool. The thematic analysis of the data was driven by the framework approach [30]. First, members of the research team conducted independent coding of the data. During several research team meetings, independent codes were then cross-checked to facilitate the development of themes, moving towards an overall interpretation of the data. Analytic rigour was enhanced by searching for negative, atypical and contradicting or conflicting cases in coding and theme development. Inter-rater reliability was ensured by integrating research team members in the final analysis, including a medical oncologist.

## Results

### The importance of distinction, professional reinvention and “keeping up”

The participants recounted many of the ‘normal pressures’ of a medical career, and medical oncology more specifically, and accepted that a universal characteristic of a medical career is competitiveness. Yet, they also noted a range of dynamics that were relatively recent, and some specific to medical oncology, which were shifting the balance of pressures in the profession. The core dynamic within this group of participants was multifaceted and centred on: the drive to distinction, largely through gaining substantive research credentials or higher qualifications; the paucity of jobs and opportunities; and, the constant reviewing and questioning of oneself in order to “keep up”. Table 2 presents some indicative quotations from the interviews.

**Table 2:** Indicative quotations: The importance of distinction, professional reinvention and “keeping up”.

Participant	Indicative quotation
#11 [female, advanced trainee, city]	We're sort of at the bottleneck …That you get through medical school, you do your internship residency, you get through and you don't fail, and that's fine, and then do medical oncology, do your physician training … it kind of takes over your life, and you just think, “I just need to finish my exams and get onto medical advanced training” … and then you find yourself in a medical oncology advanced training position, then you're like, ooh, everyone's talking about there being no job, and you just think, "Gosh, I’ve just put myself through the wringer for the last ten years of medical school and then physician exams and everything and then like, you mean there's more hoops to jump through and it’s like smaller hoops, and higher, and less hoops for people to fit through?" You just think, "Oh my god, it doesn’t end."
#22 [female, advanced trainee, city]	I know there’s a constant pressure to do research and … if you don’t do research, you’re not going to go far, you’re not going to go anywhere, that’s like a feeling that I get, I suppose. People say, because it’s so competitive these days to get jobs, you need to be on top of your game, doing research, writing things, publishing things and. . . but I'm not, I don’t feel like I’m naturally a researcher … I think research should always be a part of somebody’s practice, I just don’t know how it quite fits in with mine yet.
#16 [male, senior consultant, city]	It’s a moving target [to be an effective medical oncologist], so you need to be constantly reviewing yourself and keep up with changes, but at same time, you can’t always keep up with changes… You need to be dynamic, reviewing how you do things.
#13 [female, early-career consultant, city]	Everyone knows you need to do *something else*. So I think research is very important…in terms of contributing to the body of knowledge, getting better therapies out there, etcetera, etcetera, etcetera. But then from a trainee perspective, or a young oncologist perspective, there are no jobs for starters.
#21 [male, early-career consultant, city]	When you look at recent appointments to hospitals, in public hospitals in Sydney, it's really a requirement now that you have higher level postgraduate degree and, really, PhD, I would imagine, is pretty much a given to be considered for a Sydney metropolitan teaching hospital position. I would think a PhD nowadays is pretty much a given. Alternative to that, maybe something like a very specialised fellowship either nationally or internationally that gave you the skills to bring something new to a department.

While the advanced trainees were particularly aware of the above criteria (i.e. distinction-reviewing-questioning) to succeed in the profession, the early-career consultants and senior consultants were also acutely aware of implications of the *cascading competitiveness* and need for distinction themselves and for their advanced trainees. The ‘professional contract’, albeit largely unspoken and certainly informal, was that there were (albeit competitive) career opportunities and training options available. Often there was a dual dynamic of a growing awareness of the *heightened competitiveness*–and thus need for distinction, reinvention–and *diminishing opportunities*. Both senior and junior participants reflected on the shifting landscape of career opportunities and the disjunctions between need (patient-based) and opportunities (career-based) in medical oncology. The majority of participants emphasised the sense of a professional “bottleneck”, reflecting a discontent around the diminishing returns from the medical oncology career trajectory, and the disjunction between community need (for cancer care) and service size and opportunities (services not keeping up with expanding patient base). This was often articulated in terms of an overwhelming sense of disappointment and/or disenfranchisement with the state of cancer services, and also, professional opportunities. This interplayed with concerns around, and the desire for, career-based and personal mentoring within a medical oncology career.

### On mentors and role models: debriefing, feedback and the “two-way event”

The aforementioned concerns around career opportunities, progression and the ‘rite of passage’ in medical oncology (and medicine more broadly) were enmeshed within, and either compounded or ameliorated by, opportunities for mentorship. The participants articulated the dual emphases on the *critical nature* of mentoring and the *regular absence* within everyday work, as illustrated by the indicative quotations shown in Table 3.

**Table 3:** Indicative quotations: On mentors and role models: Debriefing, feedback and the “two-way event”.

Participant	Indicative quotation
#7 [male, senior consultant, city]	So mentorship is a two-way event and it happens because you, the mentee, really respect somebody and you try and affiliate and engage with that person and learn from them, and the mentor also decides they trust you, and respect you, and want to help you be more successful in your career. Yeah, it’s a two-way thing.
#4 [female, senior consultant, city]	I’ve mentored a lot of people and my own experience has taught me how important it is ‘cause I was wanting that during my training. I know that feeling of wanting that and not getting it, and I’m very careful when I sometimes see junior people and I think, “Oh, they remind me of me”.
#15 [male, advanced trainee, city]	There’s not very many clear avenues of people that we can talk to about when there are difficult situations or really any set ways of referral to somebody who may have a debriefing strategy for us. It just doesn’t exist as far as I’m aware … I certainly didn’t have that level of support and that is something which has the ability to affect your mental health in a detrimental way … in the absence of a clearly supportive way to do that [debrief], I feel it still becomes difficult or it still becomes hard for any individual to admit that they’re struggling with something because that may come across as a form of weakness.
#2 [male, early-career consultant, regional]	It’s one of those cultural um barriers … everyone’s busy and yeah, there never seems to be the right moment or opportunity to sit down and say, “Geez, I’m struggling with this. What sort of things could you recommend?” … nothing’s in the timetable, there’s no dedicated slot to talk about these things and so I guess you always just feel like, and you know your bosses are really busy and you just don’t wanna pester them about stuff that’s not directly patient care.
#11 [female, advanced trainee, city]	It's also doing things and stuffing up and going and saying, "Oh god, I should not have said that. That was such a bad way of saying it. Next time this sort of patient comes in, I'm got to say something else."
#19 [female, senior consultant, city]	You can come out of [a consultation] feeling completely drained and exhausted by it … Somehow, when you go home, you've got just to switch off from the situation … You have to somehow distance yourself from it. I think debriefing at the end of it with a colleague is usually really helpful just to talk about, you know, could things have been done differently or what was good about what happened and then trying to move on.

Related to mentoring were accounts of the significance of working environments that afforded opportunity to debrief with, and gain feedback or advice from, colleagues on a day-to-day basis. A key theme discussed in the interviews was the lack of opportunities for disclosure of weaknesses and vulnerabilities, and a sense of unreasonable expectations from others. This was often articulated as a lack of opportunity to learn or grow from experiences [22]. Rather than providing a space for disclosure, the professional milieu (of medicine more broadly, and medical oncology more specifically) was often talked about as *silencing struggle* and as one participated stated, treating oncologists as “bullet-proof, [and that] you can cope with anything that gets thrown at you”. While some degree of emotional self-management was accepted as normal for a profession such as medical oncology, the concern amongst the participants centred on the loss of learning opportunities through collegiality and willingness to reveal ‘mistakes’.

The significance of mentors and role models, along with a professional/institutional culture of ‘active’ debriefing and feedback, was particularly relevant for female participants, given the awareness across the participant group of increasing female representation within the workforce, and the benefits and challenges of oncology therein.

### The feminisation of the workforce and gendered pathways

One of the key themes that emerged from the interviews was the extent to which medical oncology offered a supportive environment for women. There was a perception within the participant group of a feminisation of the medical oncology workforce (i.e. greater number of female trainees). For the female participants, other female role models clearly provided strong motivation for career development. For the male participants, the challenges faced by their female counterparts were well-understood. Some of the female participants reflected in particular on the dynamics of mentoring from a gendered perspective, and the importance of maintaining connections, and receiving input from senior women (see [32, 33]). Moreover, several of the male participants also flagged the challenges faced by the workforce in sustaining successful careers for female medical oncologists. As the excerpts in Table 4 indicate, several participants recognised significant challenges for women in oncology (and medicine more broadly). While some participants articulated the structural disadvantages experienced by many female medical oncologists, others signalled medical oncology as a specialty area that was improving in terms of flexibility for the female workforce, and was more accepting and accommodating of women. However, it was clear from the interviews that female medical oncologists should be better supported. Support through mentoring, and the value therein, was then partnered with a concern for better outcome for patients (and professionals) through processes of learning and feedback.

**Table 4:** Indicative quotations: The feminisation of the workforce and gendered pathways.

Participant	Indicative quotation
#17 [female, advanced trainee, city]	I think that in oncology that there is increasing acceptance of women, part-time women, childbearing women [laughs] because you’re getting a lot of women in oncology and they are at that age because you’ve done a postgraduate degree where, actually they’re in their 30s and this is the time you think about having kids and so therefore it’s an issue and I think that my impression is that it’s dealt with relatively, increasingly better in oncology or it's, there's a realisation that, “Look, we have to be accommodating for this,”
#13 [female, early-career consultant, city]	I also was thinking I want to have a family at some stage and that oncology from a lot of my supervisors and more senior colleagues, a lot of them were women who had managed to balance family and a career and so that seemed very attractive to me as well. They work bloody hard for it [laughs] but they have managed to have it all and so that's what I thought as well … In my department I think, there's probably six women I think but those women are all really good role models and many of them are friends now. I think that that's really invaluable so that helps you to kind of think about the pathway you want to take or who you're most like and who you want to take the opinion of more.
#19 [female, senior consultant, city]	I mean certainly, many of my colleagues would, your female colleagues, if they’re trying to work out where do they have a family in their career path would go and choose a very successful medical oncologist who’s managed to juggle their clinical practice, their research, having a family.
#10 [male, senior consultant, city]	I’d just like to specifically touch on the issue of women because I’ve trained a lot of female medical oncologists and they, like, women in medicine in general, they face very, very special challenges … women make up a large proportion of the training pool but they, by the time you get to the upper levels of academic leadership, there are very few of them. They’re grossly underrepresented in academic medicine in general but particularly so in medical oncology, and I think that’s a serious problem and it’s a serious issue for the workforce … The men have a massive advantage because they don’t have to take a couple of years off to raise children … that’s an imbalance that they don’t have and it happens at a very critical point in, in career development because in comparison to their sisters, they have an advantage in also being able to go overseas and do postdoctoral fellowships and whatever and jump rungs up the ladder that their sisters are denied.
#7 [male, senior consultant, city]	What about the young oncologist, the feminisation of the workforce and how to deal with that and provide opportunities so that women can stay in the workforce and re-enter the workforce? It’s a whole lot of very practical questions that I think need answering.

### The emotional work of oncology: The intimacy-detachment tension

The interviewees regularly emphasised the inherent challenges from a professional practice, and career sustainability level, of *managing emotions* at work (see also [34]). Often this was set up as a tension between the *importance of intimacy* versus the *art of detachment*. As shown in the excerpts in Table 5, there was an acute sense of the impact of affect within oncological work, but of emotions as central to the delivery of authentic care. Throughout the interviews we heard accounts of the balancing act between ‘over’ and ‘under’ investing emotionally in oncology practice, patients and families.

**Table 5:** Indicative quotations: The emotional work of oncology: The intimacy-detachment tension.

Participant	Indicative quotation
#4 [female, senior consultant, city]	It’s exhausting um but … time heals. So you’ll feel it more emotionally acutely while it’s happening and it hurts, it *physically hurts*. Watching this man [who is dying] physically hurts me and my nurse. We’re in pain physically because of the emotional strain of watching a person suffer.
#3 [male, early-career consultant, city]	So the intrinsic things, that is the ability to philosophically analyse the meaning of life and the meaning of suffering and the meaning of the role in dealing with those things because medical oncologists usually have, I mean, this is our job … You can’t fall into a heap each time your patient dies or something like that and you can’t also be immune from it because I think you’ll have difficulty, or patients have difficulty trusting you if they don’t feel that you are actually on their side. So it’s a very interesting juggling act.
#15 [male, advanced trainee, city]	If you weren’t affected by that [seeing patient deteriorate] then you wouldn’t be having the strength of the relationship that you need … I think that actually would be a strength within somebody to have that level of emotional connection to their patients and I wouldn’t want to not have that connection but it does, of course, lead to difficulty with any time that situation arises.
#13 [female, early-career consultant, city]	From a personal perspective, dealing with death and dying sometimes can be really difficult and so I think it's about making sure you're very balanced, making sure you debrief, making sure you do other things, not just oncology. Otherwise, you wouldn't survive I think… [being] around a dying person … it's something that is quite humbling and is a bit of a reminder for me how to live my life. . .
#6 [male, senior consultant, city]	Advanced communication and reflection about mortality, and about values, and about meaningful communication with other human beings are characteristics that are transformative for you personally. So I think that's a very powerful influence on the profession when done well. A lifetime of reflection on that process is, does good things for you, I think, and changes you.
#1 [male, early-career consultant, regional]	Unfortunately, I’ve got patients queuing up at the door, I’ve got to see 25 patients today, and it’s [emotional engagement] just not possible. So I often, the shortcut is to, particularly with those who have got unresolved emotional issues, is to divert that to some colleagues, so either nursing or social work colleagues.

Linking back to issues around debriefing, the participants recounted a well-documented challenge within medical oncology in terms of managing the affective dimensions of professional work, and the tensions between interpersonal authenticity and survival. With statements like “have to learn to put up a barrier” or “I’m not here to save their lives” or, “I’m here because they want me for my particular skill”, we see the ways in which the participants embark on emotional boundary work as professional survival. For others interviewed here, this *intimate tussle* reflected a core quality of the profession–and a site of personal and professional growth and uniqueness that drew people to the profession.

### “Okay, chemotherapy for you, next patient, next patient”: Volume, necessity and service sustainability

A final key theme–and one that intersects with each of the issues outlined previously–was patient volume in oncology. Whilst medicine more broadly has become increasingly time-pressured and intensified, the volume of cancer patients, and particularly those requiring treatment by medical oncologists, was viewed by participants as increasing significantly in recent years, with a flow-on effect of added pressure (enhanced by lack of growth in services, relative to patient numbers). The demands, across the interviews were talked about as requiring “cutting corners” and diminishing capacity to engage in *self-sustaining activities*.

The service pressure issues outlined in Table 6 were articulated as inseparable from the qualities of care. That is, they dictated the “sort of care” that patients received. Whether related to patience, capacity to “feel”, willingness to listen, or avoidance of burnout, unsustainable patient numbers was seen as fundamentally challenging the practice and character of the workforce. The disjuncture between performing medical oncology work in ways that are subjectively meaningful and ways to meet structural service demands produced tensions in the day-to-day practice. Moreover, questions are raised pertaining to not only service sustainability with diminishing resources but also the effectiveness of medical oncologists when they are unable to provide the necessary clinical care–a disconnect between what one has to do and what one wants to do.

**Table 6:** Indicative quotations: “Okay, chemotherapy for you, next patient, next patient”: Volume, necessity and service sustainability.

Participant	Indicative quotation
#1 [male, early-career consultant, regional]	I came into this area because of the love of the clinical work and I like seeing patients, but it becomes that my ability to do so in a safe and effective manner is being hampered by hospital bureaucracies … My biggest concern is the large number of patients that I see and it then becomes very difficult to keep on top of all of them and take the time out to discuss them and digest it all… When you’re rushing things through, it means that you’re always cutting corners and there’s risks that you’re not providing the best holistic care that you otherwise could. I really think to be an effective oncologist I think you need to be able to commit that time in order to be doing an effective job and if it becomes a box ticking exercise, “Okay, chemotherapy for you, next patient, next patient,” it dehumanises the relationship
#2 [male, early-career consultant, regional]	According to the Medical Oncology Guidelines of Australia, the safe practice numbers you know, we see probably 60 or 70% more than what the recommendation is … I just feel like, there’s no way I can get a patient of mine, you know potential patient of mine to say, “well look sorry, I’m too busy for you” I mean the balance is between what you want to do as an oncologist and what you’re allowed to do are an issue, a day-to-day issue, no question.
#6 [male, senior consultant, city]	Time is the most important thing. I think it's the time-volume relationship that you can't provide that sort of care to 600 patients a year … because of the necessity for reflection and recovery … If you're skating on the edge of burnout, then it is very difficult to have patience with patients and their families. If you've got a limited stock of patience, if the executives have exhausted it by the time you see your first patient, you're not going to have that reserve and resilience, so I think how much patience we have with families and demands of patients, et cetera, is affected by how much reserve you've got after you've dealt with the system.
#8 [male, senior consultant, regional]	Unfortunately, the workload in the public hospital is such that they’re still seeing their 70 patients a week, as well as we are, 70 consultations anyway, usually they’re individual patients, and then busy, stretched clinics so, so I think the environment does shape the way we pract. . . practice, the way we approach our medicine in that regard.
#22 [female, advanced trainee, city]	It’s difficult but I mean we manage but it’s difficult because the demands of a clinic, being double or triple booked, running until 6 or 7 o'clock in the evening, always home very late and then up the next day in the morning. It’s really tough. And then to have to deal with ward calls and consults when you’re actually managing a very busy clinic, when you’re already running an hour behind, I sometimes get quite overwhelmed.

We acknowledge that many of the issues outlined in the identified themes are challenges across medicine and not specific to medical oncology. However, there are also unique challenges within this practice setting, a burgeoning patient population, and a professional “bottleneck” as services are not expanded to meet demand within the Australian context (and in other international settings). Our interviews revealed the complex nexus of relationships, service structures, values, care and career mapping.

## Discussion

### A time of change

Previous scholarship within the social sciences, health and medicine illustrate the importance of recognising the interplay of workforce concerns with capacity to deliver quality oncological care, and ultimately, albeit indirectly, therapeutic outcomes (e.g., [35–39]). Thus, as workplace structures shift, career pathways evolve, and work practices change, it is important to examine the nexus of professional structures and everyday practice. The findings from this study show that these medical oncologists in particular, and potentially the broader profession, are facing new and significant challenges to the way they perceive their profession, career pathways, and the delivery of care.

### Workforce structures versus professional values

Many of the new demands within the profession–to work smarter, better or simply more economically–were positioned as degrading the capacity to deliver and enact the core principles of oncology care, and moreover, were presented as a threat in sustaining the intensity and emotional work involved in care of cancer patients. The notion of *professional reinvention*, for example, as articulated by the participants reflected an increasingly pressurised and precarious professional environment they existed within; a professional structure viewed as having the potential to undermine the values that made medical oncology appealing as an original career path. The impact of *intensification*, in turn impacted their value-driven imperatives (cf. [40])–whether they could practise in such a way that sustained their own vision of the art and science of oncological work. The particular attributes of medical oncology, which place the practitioner at the centre of care in a complex treatment paradigm is threatened by the rising volume of patients and time limited treatment schedules that threaten job satisfaction.

### A time of professional uncertainty

Uncertain professional futures, and a narrowing of opportunities, heighten the pressure on achieving more with less, and juxtapose patient/community needs with professional realities. It places medical oncologists in the position of having to compete with other oncologists in a labour market that consigns professional burden on top of the emotional work they need to perform. While *professional reinvention* can in many contexts be a positive force (professional development, change, avoidance of burnout or fatigue), it can have negative implications if uncertainty is enmeshed with anxiety. If *professional reinvention* is positioned as one sided, reflecting only the needs of structures, the practitioner becomes an adjunct to the practice rather than the centre of the practice. This can undermine their confidence–if not in themselves then in their profession.

### Profession/peer support networks and sustainability

It is no coincidence that in a context of intensification and precariousness, the participants raised *mentoring* and *workplace debriefing* as crucial determinants of a successful or tolerable career in oncology. As a consequence of the shift in perspective in the way job opportunities are viewed, security and work practices, the desirability of effective *peer networks* and support through *mentorships* and feedback loops is now being articulated. The participants emphasised the need for–but often lack of–shared cultures where debriefing and feedback are part of ongoing learning.

This had strong motivational rewards particularly from a gendered position for female participants. However, the value of support through mentoring transcends gender as it was talked about and considered to lead to better outcomes for patients through dynamic learning. The study findings suggest that a shift from individual ways of working to a collective approach of sharing knowledge, experience and concerns is actively being positioned as a desirable outcome. From this contextual perspective, mentoring and collegial approaches to addressing work pressure ameliorate the sense of isolation when a situation such as meeting patient load targets seems to be unreasonable.

### Managing expectations: workload versus patient needs

The interviews illustrate the acute challenge of emotion management within professional life and workforce sustainability, and the considerable individual, interpersonal and professional challenges that this produces. Indeed, the emotional boundaries and tensions of service delivery and care in medical oncology, and medicine more broadly, are well documented (e.g., [41–44]). These results also remind us that such processes cannot be separated from the aforementioned structural shifts, mentoring dynamics, debriefing practices and so forth. That is, the structural environment operates in conjunction with micro interactions and care practices of medical oncologists. Thus, professional workload issues, volume, burnout and so forth each articulate both the burden of emotions but also the expectations on the profession as a whole (and as an evolving entity).

There is considerable concern here that increased patient volume and intensification–also shown in other studies–will lead to poorer outcomes for both themselves (e.g. burnout), and their patients in terms of the quality of care and support they expect from their medical oncologists. Resorting to a purely disease focus, rather than person-centred approach, becomes the ultimate implication of such pressures [45]. The individual medical oncologist is thus having to make difficult choices in relation to these structural changes to the workforce/service delivery that does not necessarily take into account individual needs and practices and the requirements of care. Burnout–a shifting of responsibility from system to individual in many cases–reflected for many of the participants the untenable nature of new work paradigms.

### The new landscape of medical oncology

Each of the issues emergent from the interviews–intensification, precariousness, emotional management, paucity of mentoring, reduction in time-with-patient, workload stress, structural change, and uncertain professional futures–have the potential to compromise care, and undermine the viability of the workforce satisfaction and the fostering of successful career medical oncologists. New dynamics–rise of patient numbers and treatment volume intensification, feminisation of the workforce, fractionised work–are compounding entrenched issues such as burnout and emotional fatigue, creating a new and challenging landscape for oncologists in Australia and internationally.

It is no longer viable to consider workforce issues in isolation and to problematise individual trajectories when the issues experienced are reflective of wider systemic/structural dynamics. The *uncertainty gap* between identifying a problem and finding a solution is experienced by many of the study participants. Understanding the day-to-day structural as well as the evident clinical challenges that come with working in the profession is foundational to meeting the needs of medical oncologists.

This study has various limitations. Our sample of 22 medical oncologists, while appropriate in size for a qualitative study, only captures the experiences of a self-selected group of oncologists, in one Australian state. As such, despite providing indications of themes and theoretical insights likely to resonate across other contexts, our findings cannot be transferred to other settings. In addition, our study did not assess the extent to which workload (and associated pressures) was increasing across and within the participant group. Thus, while it was clear from the interviews that participants perceived their workload to be increasing, we cannot make claims, based on our findings, related to broad structural increases in patient numbers or treatment volume intensification. Further research focused on the ways by which the workload of medical oncologists may be increasing, including those within various subspecialties, locations, and across public and private practice, is needed to better understand the experiences of the workforce.

## References

[1] Band PR. The birth of the subspecialty of medical oncology and examples of its early scientific foundations. Journal of Clinical Oncology. 2010;28(22):3653–8. 10.1200/jco.2010.29.5261 20567013

[2] Olver I. Memories of MOGA. Asia-Pacific Journal of Clinical Oncology. 2009;5(Suppl 1):A1–A18. 10.1111/j.1743-7563.2009.01207.x 31381829

[3] Popescu RA, Schäfer R, Califano R, Eckert R, Coleman R, Douillard J-Y, et al The current and future role of the medical oncologist in the professional care for cancer patients: a position paper by the European Society for Medical Oncology (ESMO). Annals of Oncology. 2014;25(1):9–15. 10.1093/annonc/mdt522 24335854

[4] Erikson C, Salsberg E, Forte G, Bruinooge S, Goldstein M. Future supply and dmand for oncologists: Challenges to assuring access to oncology services. Journal of Oncology Practice. 2007;3(2):79–86. 10.1200/jop.0723601 20859376 PMC2793740

[5] Hortobagyi GN. A shortage of oncologists? The American Society of Clinical Oncology workforce study. Journal of Clinical Oncology. 2007;25(12):1468–9. 10.1200/JCO.2007.10.9397 17360965

[6] Australian Medical Workforce Advisory Committee. The specialist medical and haematological oncology workforce in Australia: Supply, requirements and projections 2001–2011. Sydney: AMWAC, 2001 Contract No.: AMWAC Report 2001.2.

[7] Blinman PL, Grimison P, Barton MB, Crossing S, Walpole ET, Wong N, et al The shortage of medical oncologists: the Australian Medical Oncologist Workforce Study. Med J Aust. 2012;196(1):58–61. . 22256937 10.5694/mja11.10363

[8] Koczwara B, Barton MB, Walpole ET, Grimison P, Blinman PL, Crossing S, et al Workforce shortages in medical oncology: A looming threat to quality cancer care. Med J Aust. 2012;196(1):32–3. 10.5694/mja11.10356 22256925

[9] Bidwell S, Simpson A, Sullivan R, Robinson B, Thomas W, Jackson C, et al A workforce survey of New Zealand medical oncologists. The New Zealand Medical Journal. 2013;126(1371):45–53. 23793120

[10] Boutayeb S, Taleb A, Belbaraka R, Ismaili N, Berrada N, Allam W, et al The practice of medical oncology in Morocco: The national study of the Moroccan group of trialist in medical oncology (EVA-Onco). ISRN Oncol. 2013;2013:341565 10.1155/2013/341565 24223311 PMC3817705

[11] de Azambuja E, Ameye L, Paesmans M, Zielinski CC, Piccart-Gebhart M, Preusser M. The landscape of medical oncology in Europe by 2020. Annals of Oncology. 2014;25(2):525–8. 10.1093/annonc/mdt559 24425791

[12] Draper C, Louw G. Choosing a career in medicine: The motivations of medical students from the University of Cape Town. Education for Primary Care. 2007;18(3):338–45.

[13] Loriot Y, Albiges-Sauvin L, Dionysopoulos D, Bouyon-Monteau A, Boyle H, You B, et al Why do residents choose the medical oncology specialty? Implications for future recruitment—results of the 2007 French Association of Residents in Oncology (AERIO) Survey. Annals of Oncology. 2009 10.1093/annonc/mdp294 19628567

[14] Ogbonna E, Harris LC. Work intensification and emotional labour among UK university lecturers: An exploratory study. Organization Studies. 2004;25(7):1185–203. 10.1177/0170840604046315

[15] Tadajewski M. Academic labour, journal ranking lists and the politics of knowledge production in marketing. Journal of Marketing Management. 2016;32(1–2):1–18. 10.1080/0267257X.2015.1120508

[16] Augustin ID, Long TR, Rose SH, Wass CT. Recruitment of house staff into anesthesiology: a longitudinal evaluation of factors responsible for selecting a career in anesthesiology and an individual training program. Journal of Clinical Anesthesia. 2014;26(2):91–105. 10.1016/j.jclinane.2013.01.020 24657015

[17] Celenza A, Bharath J, Scop J. Improving the attractiveness of an emergency medicine career to medical students: An exploratory study. EMA—Emergency Medicine Australasia. 2012;24(6):625–33. 10.1111/j.1742-6723.2012.01607.x 23216723

[18] Burchell B, Ladipo D, Wilkinson F. Job insecurity and work intensification London & New York: Routledge; 2005.

[19] Kuhlmann E. Modernising health care: Reinventing professions, the state and the public Bristol: Policy Press; 2006.

[20] Sennett R. The corrosion of character: The personal consequences of work in the new capitalism New York: WW Norton & Company; 2011.

[21] Stokes H, Wyn J. Constructing identities and making careers: Young people’s perspectives on work and learning. International Journal of Lifelong Education. 2007;26(5):495–511. 10.1080/02601370701559573

[22] Collinson DL. Identities and insecurities: Selves at work. Organization. 2003;10(3):527–47. 10.1177/13505084030103010

[23] Rubin G, Berendsen A, Crawford SM, Dommett R, Earle C, Emery J, et al The expanding role of primary care in cancer control. The Lancet Oncology. 16(12):1231–72. 10.1016/S1470-2045(15)00205-3 26431866

[24] Gordon R, Eagar K, Currow D, Green J. Current funding and financing issues in the Australian hospice and palliative care sector. Journal of Pain and Symptom Management. 2009;38(1):68–74. 10.1016/j.jpainsymman.2009.04.002 19615629

[25] Edwards N, Kornacki MJ, Silversin J. Unhappy doctors: what are the causes and what can be done? BMJ (Clinical research ed). 2002;324(7341):835–8. Epub 2002/04/06. 11934779 10.1136/bmj.324.7341.835 PMC1122769

[26] Girgis A, Hansen V, Goldstein D. Are Australian oncology health professionals burning out? A view from the trenches. European Journal of Cancer. 2009;45(3):393–9. 10.1016/j.ejca.2008.09.029 19013790

[27] Levit L, Smith AP, Benz EJ, Ferrell B. Ensuring quality cancer care through the oncology workforce. Journal of Oncology Practice. 2010;6(1):7–11. 10.1200/jop.091067 20539724 PMC2805353

[28] ABS. 1216.0—Australian Standard Geographical Classification (ASGC) Canberra: Australian Bureau of Statistics; 2010 [cited 2015 15 December]. Available from: http://www.abs.gov.au/AUSSTATS/abs@.nsf/Lookup/1216.0Main+Features 1July 2010?OpenDocument.

[29] Bryman A. Social Research Methods 4th ed New York: Oxford University Press; 2012.

[30] Pope C, Mays N. Qualitative methods in health research In: Pope C, Mays N, editors. Qualitative Research in Health Care. UK: Blackwell; 2006 p. 1–11.

[31] Australian Government DoH. Australian Standard Georgraphical Classification—Remoteness Area (ASGC-RA) [cited 2016 April 15]. Available from: http://www.health.gov.au/internet/otd/Publishing.nsf/Content/RA-intro.

[32] Hilmer C, Hilmer M. Women helping women, men helping women? Same-Gender mentoring, initial job placements, and early career publishing success for economics PhDs. The American Economic Review. 2007;97(2):422–6.

[33] Levinson W, Kaufman K, Clark B, Tolle SW. Mentors and role models for women in academic medicine. Western Journal of Medicine. 1991;154(4):423–6. PubMed PMID: PMC1002790. 1877183 PMC1002790

[34] Nettleton S, Burrows R, Watt I. How do you feel doctor? An analysis of emotional aspects of routine professional medical work. Social Theory & Health. 2008;6(1):18–36. 10.1057/palgrave.sth.8700112

[35] Nancarrow SA, Borthwick AM. Dynamic professional boundaries in the healthcare workforce. Sociology of Health & Illness. 2005;27(7):897–919. 10.1111/j.1467-9566.2005.00463.x 16313522

[36] Oh H. Hospital consultations and jurisdiction over patients: consequences for the medical profession. Sociology of Health & Illness. 2014;36(4):580–95. 10.1111/1467-9566.12087 24963531

[37] Yoon JD, Daley BM, Curlin FA. The association between a sense of calling and physician well-being: A national study of primary care physicians and psychiatrists. Academic Psychiatry. 2016:1–7. 26809782 10.1007/s40596-016-0487-1

[38] Shanafelt T, Raymond M, Horn L, Moynihan T, Collichio F, Chew H, et al Oncology fellows' career plans, expectations, and well-being: Do fellows know what they are getting into? Journal of Clinical Oncology. 2014;32(27):2991–7. 10.1200/jco.2014.56.2827 25049326 PMC4876315

[39] Shanafelt T. Finding meaning, balance, and personal satisfaction in the practice of oncology. Journal of Supportive Oncology. 2005;3(2):157–62, 64. Epub 2005/03/31. . 15796448

[40] Miles A. On the interface between science, medicine, faith and values in the individualization of clinical practice: a review and analysis of 'Medicine of the Person' Cox J., Campbell A. V. & Fulford K. W. M., eds (2007). Journal of evaluation in clinical practice. 2009;15(6):1000–24. Epub 2010/04/07. 10.1111/j.1365-2753.2009.01351.x . 20367700

[41] Baile WF. Giving bad news. The Oncologist. 2015;20(8):852–3. 10.1634/theoncologist.2015-0250 26185197 PMC4524764

[42] Bascioni R, Giorgi F, Esperide B, Brugni M, Basirat F, Rastelli F, et al Medical oncologist's commitment in end-of-life care of cancer patients. Palliative & Supportive Care. 2014;12(5):351–4. 10.1017/S1478951513000291 23768912

[43] Fallowfield L, Guarneri V, Ozturk MA, May S, Jenkins V. Blurring of boundaries in the doctor-patient relationship. The Lancet Oncology. 2014;15(13):1423–4. Epub 2014/12/03. 10.1016/s1470-2045(14)71122-2 . 25456361

[44] Broom A, Kirby E, Good P, Wootton J, Adams J. The art of letting go: Referral to palliative care and its discontents. Social Science & Medicine. 2013;78:9–16. 10.1016/j.socscimed.2012.11.008 23219848

[45] Jacob SA, Ng WL, Do V. Estimation of an optimal chemotherapy utilisation rate for cancer: Setting an evidence-based benchmark for quality cancer care. Clinical Oncology. 2015;27(2):77–82. 10.1016/j.clon.2014.10.004 25455844

